# Relationships between physical activity and muscular strength among healthy adults across the lifespan

**DOI:** 10.1186/s40064-015-1357-0

**Published:** 2015-09-28

**Authors:** Allie Leblanc, Linda S. Pescatello, Beth A. Taylor, Jeffrey A. Capizzi, Priscilla M. Clarkson, C. Michael White, Paul D. Thompson

**Affiliations:** University of Connecticut, 2095 Hillside Rd Unit 1110, Storrs, CT 06269 USA; University of Massachusetts, 110 Totmann Building, 30 Eastman Lane, Amherst, MA 01003 USA; Division of Cardiology, Henry Low Heart Center, Hartford Hospital, 80 Seymour St, Hartford, CT 06102 USA; 31 Silversmith Rd, Unionville, CT 06085 USA

**Keywords:** Accelerometer, Isokinetic dynamometer, Physical activity, Muscle strength, Classification, Methods, Aging

## Abstract

The purpose of this study was to examine relationships between objective and self-report measures of physical activity and muscle strength among healthy adults ranging in age from 20 to 91 years. Participants (n = 412) were mostly Caucasian men (48 %) and women (52 %) 43.9 ± 16.1 year of age with a body mass index (BMI) of 26.4 ± 4.8 kg/m^2^. Physical activity was measured objectively with an accelerometer and by self-report with the Paffenbarger Physical Activity Questionnaire. Upper and lower body muscle strength were measured with an isokinetic dynamometer and handgrip strength with a static dynamometer. Multivariate regression assessed relationships between physical activity and muscle strength. The strongest correlates of upper body strength including handgrip strength were gender (r = −0.861 to −0.716), age (r = −0.445 to −0.241), BMI (r = 0.134–0.397), and physical activity (r = 0.093–0.186). The strongest correlates of lower body strength were gender (r = −0.772 to −0.634), age (r = −0.663 to −0.445), BMI (r = 0.160–0.266), and physical activity (r = −0.139 to 0.151). The strongest correlates of muscle strength were gender (explaining 40–74 % of the variance), age (6–44 %), and BMI (2–16 %), while physical activity correlations were weaker (1–3 %). Conflict surrounding the influence of a physically active lifestyle on muscle strength with age may be due to the stronger influences of other factors that supersede those of physical activity whether measured objectively or by self-report methods.

## Background

Regular participation in physical activity promotes healthy weight, bone mass, and muscle function as well as prevents falls and fractures in older adults. These and other numerous health benefits ultimately extend active life expectancy (USA Department of Health and Human Services 2008). Physical inactivity is a major determinant of the loss of muscle strength as is gender, aging, and body mass index (BMI) (Hollmann, Struder, Tagarakis, and King 2007; Hortobagyi, Katch, Katch, LaChance, and Behnke 1990; Musselman and Brouwer 2005). Maximum muscle strength is typically achieved between 20 and 30 year (Bosco and Komi 1980) and begins to decline around an age of 40 year (Kallman, Plato, and Tobin 1990). After 70 year, overall muscle strength declines 3.6 % annually for men and 2.8 % for women (Goodpaster et al. 2006).

Nonetheless, the literature on relationship between physical activity and muscle strength as modulated by age is mixed. Utilizing a self-report physical activity questionnaire, (Paalanne et al. 2009) found greater maximal isometric trunk muscle strength measured by a computerized dynamometer among 874 healthy, young men and women with high levels of moderate to vigorous intensity physical activity compared to those with lower levels of physical exertion. Rantanen et al. (1997) found physical activity assessed by questionnaire positively associated with maximal isometric strength of several muscle groups evaluated by an dynamometer among 287 older adults.

Sandler et al. (1991) found physical activity as assessed by the Paffenbarger physical activity questionnaire (Paffenbarger, Wing, and Hyde 1978) positively correlated with muscle strength assessed by a dynamometer among a sample of 620 middle-aged to older women. Furthermore, these investigators found physical activity to be the second largest contributor to the variance in muscle strength (r = 0.54) second to age (r = 0.48) (Sandler et al. 1991). Forrest et al. (2007) also assessed physical activity using the Paffenbarger physical activity questionnaire (Paffenbarger et al. 1978) in a sample of about 20,000 older women and found physical activity positively associated with handgrip strength measured via dynamometry. Jakobsen et al. (Jakobsen, Rask, and Kondrup, 2010) found physical activity assessed with the Baecke questionnaire positively associated with handgrip strength in women, but not men ranging in age from 25 to 65 year. In contrast, other investigators utilizing self-report physical activity questionnaires have found no correlation with physical activity and muscle strength among older populations of men and women (Bryant, Trew, Bruce, and Cheek 2007; Daly et al. 2008). Collectively, in these studies, several investigative employed self-report questionnaires that were validated (Bryant et al. 2007; Forrest et al. 2007; Jakobsen et al. 2010; Rantanen et al. 1997; Sandler et al. 1991), while others did not (Daly et al. 2008; Paalanne et al. 2009), perhaps contributing to the inconsistencies in this literature.

Adding to these divergent findings are the two studies assessing physical activity with an accelerometer. Gerdhem et al. (Gerdhem, Dencker, Ringsberg, and Akesson, 2008) found physical activity assessed with an accelerometer did not correlate with knee extension and flexion muscle strength among 57 older women. Similarly, Morie et al. (2010) found no differences in upper body and lower extremity muscle strength between the low and high physical activity groups measured by an accelerometer among 82 older men.

Reasons for the discrepancies among studies examining the relationships between physical activity and muscle strength are unclear but could reside in the methods that were used to assess physical activity and muscle strength as well as differences in the characteristics of the population studied. Previous reports (Bryant et al. 2007; Daly et al. 2008; Forrest et al. 2007; Gerdhem et al. 2008; Jakobsen et al. 2010; Morie et al. 2010; Paalanne et al. 2009; Rantanen et al. 1997; Sandler et al. 1991) have examined either self-report or objective measures of habitual physical activity and measures of muscle strength in populations with narrow age ranges and that may have included only one gender. Additionally, examination of other factors that have been documented to influence the relationship between physical activity and muscle strength within the same study is generally lacking.

Therefore, the purpose of this study was to examine the relationships among a self-report and objective measure of physical activity and muscle strength among a large sample of healthy, men and women from 20 to 91 year to provide insights into the mixed literature.

## Methods

### Experimental approaches to the problem

This sub-study derived from a larger National Institutes of Health (NIH) funded study entitled, “The Effect of Statins on Skeletal Muscle Function” (STOMP) (NIH RO1 HL081893-01A2) (P. D. Thompson et al. 2010). The specific aims of STOMP were to examine the incidence rate of statin-induced muscle pain or discomfort, also known as myalgia, and the effects of statins on muscle strength, endurance, and aerobic exercise performance in a healthy population taking 80 mg of Atorvastatin or a placebo (P. D. Thompson et al. 2010). The STOMP methods have been described in detail elsewhere (Ballard et al. 2013; Grimaldi et al. 2013; Parker et al. 2013; Stroes et al. 2015; P. D. Thompson et al. 2010). This sub-study used baseline data from STOMP to examine the relationships among objective and self-report measures of physical activity and isokinetic and isometric measures of muscular strength and endurance among a population of healthy men and women ≥20 year with no present or previous history of statin use (P. D. Thompson et al. 2010). STOMP was approved by the Institutional Review Board (IRB) of the participating sites that included Hartford Hospital, the University of Connecticut, and the University of Massachusetts Amherst.

All data collected for this sub-study were taken from STOMP study visits 1–3 (V1, V2, V3 respectively) prior to randomization to either a placebo or 80 mg of Atorvastatin (P. D. Thompson et al. 2010). All data were manually entered into an electronic website maintained by the study coordinator at Hartford Hospital.

### Subjects

A total of 220 men and 220 women ≥20 year were recruited for STOMP and were equally distributed within three designated age categories (20–39, 40–54, 55+ year) over 4 years at the three testing sites. Recruitment strategies included local and campus newspaper advertisements, flyers, and posters (P. D. Thompson et al. 2010). Once a potential subject expressed interest, they underwent a phone interview to determine eligibility based on a strict set of exclusion criteria. Participants were excluded if they were presently or had previously been treated with cholesterol-lowering medications, or had been diagnosed with diabetes mellitus, hyper- or hypothyroidism, or any heart condition that required medication or a restriction of physical activity. Anyone unable to exercise vigorously on a treadmill or who had hepatic disease, renal disease, or occult cardiac ischemia documented during a physician supervised treadmill test during STOMP V1 was also excluded from the study.

Individuals using hypertensive medications were included if they had been on these medications for at least 3 months and their blood pressure (BP) was stable (<140/90 mmHg). BP was monitored during V1 and V2 to ensure each subject’s eligibility. Women of childbearing age were given a pregnancy test at the start and conclusion of the study and were asked to use contraception throughout the duration of their participation in the study. The population for this sub-study (n = 412) was young, healthy Caucasian men (n = 198) and women (n = 214). Subjects were on average middle aged (43.9 ± 16.1 year) and overweight (26.4 ± 4.8 kg/m^2^) and had optimal blood pressure (118.9 ± 13.3/75.3 ± 9.7 mmHg). Men were heavier (27.4 ± 4.5 kg/m^2^) and had higher maximum oxygen consumption (VO_2max_) (38.3 ± 8.9 ml/kg/min) than women (25.4 ± 5.0 5 kg/m^2^, 30.0 ± 8.8 ml/kg/min respectively) (p < 0.001). Women had higher resting heart rate (70.5 ± 10.8 bpm) (p < 0.01) and lower blood pressure (116.3 ± 13.4/74.2 ± 9.8 mmHg) than men (67.3 ± 11.8 bpm, 121.8 ± 12.7/76.6 ± 9.4 mmHg respectively) (p = 0.03).

### Procedures

#### Anthropometric Measurements

This sub-study used baseline anthropometrics from V1. Body weight (kg) and height (m) were measured using a calibrated balance beam scale and a wall mounted tape measure. BMI was then calculated (kg/m^2^). Waist circumference (cm) was measured with a Gulick spring-loaded non-dispensable tape measure (P. D. Thompson et al. 2010). Subjects stood erect with their arms at their sides, feet together and abdomen relaxed, and a horizontal measurement was taken at the narrowest part of the torso above the umbilicus and below the xiphoid process.

### Muscular strength and endurance

Muscle strength and endurance were assessed on V1, V2, and V3. Visit 1 was used to familiarize the subject with the Biodex and the handgrip dynamometer. The data from V2 and V3 were used as the muscle strength measures.

#### Handgrip strength

Isometric handgrip strength was assessed on the dominant hand using a handgrip dynamometer (P. D. Thompson et al. 2010). The subject performed three maximal contractions for 3 s each, with 1 min of rest between each contraction. The average of the three contractions was used as the measure of average peak torque (Nm) (P. D. Thompson et al. 2010).

#### Lower body

All muscle strength and endurance measures were assessed using the Biodex System 3 Isokinetic Dynamometer (Biodex Medical, Shirley, NY) (P. D. Thompson et al. 2010). Before each visit, the Biodex was calibrated following the procedures outlined by Pincivero et al. (2003) (Pincivero, Campy, and Coelho, 2003). Subjects warmed up by performing two submaximal knee extension contractions at 10 %, two contractions at 50 %, and two contractions at 90 % effort (P. D. Thompson et al. 2010). Subjects were seated for all leg testing and had their arms folded across their chest and secured with Velcro straps at the thigh, pelvis, and torso to prevent extraneous movement. The lateral femoral epicondyle of the subject was aligned with the axis of rotation of the lever arm of the Biodex (P. D. Thompson et al. 2010). The dominant hand side knee was tested.

##### Isometric strength

Subjects performed three isometric contractions at a knee angle of 110°. Subjects started the test by kicking out and holding this position for 4 s followed by a 1 min rest. They then pulled back as hard as they could and held this position for 1 min followed by another 1 min rest. This procedure was repeated until the subject had completed a total of three kicks and three pulls. They then rested for 5 min. The average peak torque (Nm) of each kick and pull was averaged.

##### Isokinetic strength

Next, subjects performed five contractions at 60°/s by doing five full range of motion kicks as hard and as fast as they could, followed by another 5 min of rest. Subjects then performed five isokinetic contractions at 180°/s by doing five full range of motion kicks in the same manner. Average peak torque (Nm) was calculated over an angular displacement of 60°. A 10 min rest was given prior to the dynamic muscle endurance testing.

##### Dynamic muscle endurance

To complete the leg muscle strength assessment, participants underwent a dynamic muscle endurance test of the knee. Subjects performed 30 consecutive full range of motion maximal contractions at 180°/s (P. D. Thompson et al. 2010). Average peak torque (Nm) was measured. A fatigue index was also calculated as a measure of muscle endurance as the average peak torque of the last 5 repetitions divided by the average peak torque of the 5 highest consecutive repetitions, multiplied by 100, and then subtracted from the total of 100 (%) (P. D. Thompson et al. 2010).

#### Upper body

Upper body strength of the biceps and triceps was tested using the elbow attachment on the Biodex. Subjects were seated with their torso at 90° of hip flexion and secured by a strap at the pelvis and two straps across the torso to prevent extra movements. The arm was positioned so that the axis of the level arm coincided with the rotational axis of the elbow joint in order to assure movement of the lower arm through the sagittal plane. A wide strap crossed the biceps brachii to ensure the alignment of the subjects elbow with the axis of the lever arm during testing. The subject performed two submaximal elbow flexion contractions at 10 %, two contractions at 50 %, and two contractions at 90 % effort to warm up (P. D. Thompson et al. 2010).

##### Isometric strength

Subjects performed three isometric contractions at an elbow angle of 90°/s. Subjects started the test by pushing out and holding this position for 4 s followed by a 1 min rest. They then pulled back as hard as they could and held this position for 1 min followed by another 1 min rest. This procedure was repeated until the subject had completed three pushes and three pulls, and was then followed by 5 min of rest (P. D. Thompson et al. 2010). Average peak torque was measured (Nm).

##### Isokinetic strength

Next the subject performed four isokinetic contractions at an elbow angle of 60°/s followed by 5 min of rest. Finally, the subject performed 4 isokinetic contractions at an elbow angle of 180°/s (P. D. Thompson et al. 2010). The average peak torque (Nm) was calculated over an angular displacement of 60° (P. D. Thompson et al. 2010).

#### Physical activity

##### Accelerometer

Subjects were given an Actical physical activity accelerometer (Mini Meter, a Respironics Inc., Bend, OR) to wear on the hip for 96 consecutive hr encompassing 2 week days and 3 weekend days immediately after V2 and then collected at V3 (P. D. Thompson et al. 2010). The epoch was set at 25 s (P. D. Thompson et al. 2010). The Actical was only removed while the subject was swimming, bathing, showering, or sleeping. The data were downloaded immediately at the following study visit (V3) to ensure that 96 h of useable data were collected. Activity levels were then averaged from the 96 h and included the following measures of physical activity: activity (counts/day), energy expenditure (kcal/d), average time spent (min/d) in sedentary, light, moderate, and vigorous intensity physical activity, and steps per day. Time spent in sedentary activity was defined as <1.5 METs, light intensity physical activity was 1.5 to <3.0 METs, moderate intensity physical activity 3.0 to <6.0 METs, and vigorous intensity physical activity >6.0 METs.

##### Paffenbarger physical activity questionnaire

The Paffenbarger physical activity questionnaire was administered by research assistants at V1 (P. D. Thompson et al. 2010). This sub-study utilized data from question 8, which asked participants to estimate how many hours on a typical weekday and a typical weekend day during the past year they participated in activities of varying intensities. Each type of movement (i.e., sleeping or reclining, sitting, or engaging in light, moderate, and vigorous intensity physical activity) was assigned a metabolic equivalent task (MET) value (P. D. Thompson et al. 2010) and total time (h) spent in each type of movement was used to calculate MET*hr/wk for each subject.

### Cardiorespiratory fitness

On V2 prior to randomization, VO_2max_ (ml/kg/min) was measured using a modified Balke maximal treadmill test (American College of Sports Medicine 2013; Balke and Ware 1959; Takken et al. 2009) assessed using a breath-by-breath analysis of expired gases though the Parvomedics True One 2400 Metabolic Cart (ParvoMedics Corp, Sandy, UT) (P. D. Thompson et al. 2010). The test was terminated when one or more of the following conditions was met: the subject reported an rate of perceived exertion of 18, the subject had a respiratory exchange ratio greater than 1.1, the subject achieved their age predicted maximum heart rate, there was a plateau in VO_2_, or the subject self-terminated due to fatigue or discomfort (American College of Sports Medicine 2013).

### Statistical analyses

The Statistical Package for the Social Sciences (SPSS) Base 18.0 for Macintosh (IBM, Armok, NY) was used to calculate all the statistics. Descriptive statistics (mean ± SEM) were calculated for all study variables. Pearson product moment correlation coefficients (r) were performed to examine the relationship between muscle strength and endurance and measures of physical activity as assessed by accelerometer or by the Paffenbarger physical activity questionnaire. Multivariate regressions were performed to assess which subject characteristics and physical activity measures were predictive of muscle strength. The variance inflation factor (VIF) was used to quantify the degree of multicollinearity of predictor variables, and any variable exceeding 3.0 was removed from the model. If variables were removed from the model due to VIF >3.0, which only occurred with the accelerometer models, the variable that was retained had the strongest correlation with the measure of strength and in most instances was total energy expenditure which encompassed the variables removed. Significance was accepted at p < 0.05.

## Results

Subjects spent most (76.2 %) of their time (min/day) being sedentary, followed by time spent in light intensity physical activity (14.4 %) and moderate intensity physical activity (9.1 %), with the least amount of time spent in vigorous intensity physical activity (0.3 %) (Table 1). Study participants easily meet the recommendations for daily physical activity with an average of 131.2 min/day spent in moderate intensity physical activity (Table 1). Although there is no published standard for any of the strength measures measured in our study, compared to similar studies, our subjects approximated average muscle strength and endurance in all measures (Forrest et al. 2007; Jakobsen et al. 2010; Paalanne et al. 2009; Rantanen et al. 1997; Sandler et al. 1991), according to other comparable datasets (Forrest et al. 2007; Jakobsen et al. 2010; Paalanne et al. 2009; Rantanen et al. 1997; Sandler et al. 1991). Men were significantly stronger in every measure of muscle strength than the women (p < 0.001) (Table 2).

**Table 1:** Measures of physical activity for the total sample and by gender (Mean ± SD)

Characteristics	Total (n = 412)	Men (n = 198)	Women (n = 214)
Total steps per day	8182.4 ± 3537.1	8448.4 ± 3379.5	7934.4 ± 3668.7
Total energy expenditure (kcal/day)	624.6 ± 275.1	722.7 ± 298.9*	531.7 ± 212.9
Actical total counts (per day)	186699.7 ± 104354.9	203739.6 ± 110309.5^ψ^	170724.9 ± 95992.9
Time in sedentary activity (actical) (min/d)	1098.1 ± 99.2	1092.8 ± 99.8	1103.0 ± 98.6
Time in light activity (actical) (min/d)	206.9 ± 59.8	204.1 ± 59.3	209.6 ± 60.3
Time in moderate activity (actical) (min/d)	131.2 ± 51.7	138.7 ± 53.3^ψ^	124.1 ± 49.2
Time in vigorous activity (actical) (min/d)	3.8 ± 7.8	4.3 ± 8.6	3.3 ± 7.0
Total self reported energy expenditure (MET*hr/wk)	388.6 ± 74.8	381.9 ± 77.5	394.9 ± 71.9

Men vs. women, γ *p* < 0.05, ^ψ^ *p* < 0.01, * *p* < 0.001

**Table 2:** Measures of muscular strength and endurance for the total sample and by gender (Mean ± SD)

Measure	Total (n = 412)	Men (n = 198)	Women (n = 214)
Handgrip strength (kg)	39.1 ± 12.8	48.6 ± 10.5*	30.3 ± 7.1
Isometric knee extension (peak torque-Nm)	190.1 ± 69.8	235.6 ± 63.4*	147.7 ± 44.4
Isometric knee flexion (peak torque-Nm)	82.4 ± 30.8	103.7 ± 26.6*	62.7 ± 19.2
Isokinetic knee extension 60˚/s (peak torque-Nm)	146.9 ± 52.6	184.7 ± 44.1*	112.1 ± 31.5
Isokinetic knee flexion 60˚/s (peak torque-Nm)	76.2 ± 30.5	97.6 ± 23.4*	56.3 ± 21.5
Isokinetic knee extension 180˚/s (peak torque-Nm)	99.6 ± 38.9	127.4 ± 33.6*	74.0 ± 22.1
Isokinetic knee flexion 180˚/s (avg peak torque-Nm)	54.3 ± 22.3	70.3 ± 19.7*	39.5 ± 14.0
Knee endurance extension peak torque-Nm)	84.7 ± 31.2	108.2 ± 25.3*	63.2 ± 17.5
Knee endurance flexion (peak torque-Nm)	46.0 ± 17.8	58.8 ± 15.2*	34.1 ± 10.3
Fatigue index (% decrease)	31.0 ± 8.5	32.3 ± 8.3^ψ^	29.8 ± 8.5
Isometric elbow extension (peak torque-Nm)	44.1 ± 20.4	59.6 ± 18.1*	29.6 ± 8.1
Isometric elbow flexion (peak torque-Nm)	51.4 ± 20.2	67.9 ± 15.5*	35.9 ± 8.2
Isokinetic elbow extension 60˚/s (peak torque-Nm)	39.1 ± 14.9	51.0 ± 11.8*	28.1 ± 6.9
Isokinetic elbow flexion 60˚/s (peak torque-Nm)	34.4 ± 14.1	46.8 ± 9.2*	22.8 ± 4.8
Isokinetic elbow extension 180˚/s (avg peak torque-Nm)	32.5 ± 20.5	43.1 ± 24.9*	22.6 ± 5.7
Isokinetic elbow flexion 180˚/s (peak torque-Nm)	30.1 ± 11.4	39.7 ± 8.5*	21.2 ± 4.4

Men vs. women, γ *p* < 0.05, ^ψ^ *p* < 0.01, * *p* < 0.001

## Multivariable regression models of correlates of muscle strength and endurance

### Accelerometer

#### Lower body

Multivariate regression correlates of lower body muscle strength and endurance are displayed in Table 3. Factors accounting for 52.7 % of the variance in isometric knee extension were gender (p < 0.001), age (p < 0.000), BMI (p < 0.001), and time spent in sedentary behavior (p = 0.005). Factors accounting for 65.6 % of the variance in isokinetic knee extension at 60°/s were gender (p < 0.001), age (p < 0.001), BMI (p < 0.001), and total energy expenditure (p = 0.002). Factors accounting for 61.5 % of the variance in isokinetic knee extension at 180˚/s were gender (p < 0.001), age (p < 0.001), BMI (p = 0.001), and total energy expenditure (p = 0.001). Finally, 74.8 % of the variance in knee endurance extension was accounted for by gender (p < 0.001), age (p < 0.001), BMI (p < 0.001), and total energy expenditure (p = 0.002). Season, test site, and VO_2max_ were not significant correlates of upper and lower body muscle strength. (p > 0.05).

**Table 3:** Multivariate models of correlates of lower body muscle strength among STOMP participants (n = 412)

Muscle strength	Predictors	β	T	Partial r	r^2^	p
Isometric knee extension	Gender	−0.574	−16.363	−0.634		<0.001
	Age	−0.342	−9.919	−0.445		<0.001
	BMI	0.185	5.248	0.254		<0.001
	Time in sedentary behavior (min/day)	−0.097	−2.809	−0.139		0.005
Model summary					0.527	<0.001
Isokinetic knee extension 60°/s	Gender	−0.606	−19.295	−0.695		<0.001
	Age	−0.404	−13.560	−0.562		<0.001
	BMI	0.155	5.022	0.244		<0.001
	Total energy expenditure (kcal/day)	0.099	3.052	0.151		0.002
Model summary					0.656	<0.001
Isokinetic knee extension 180°/s	Gender	−0.618	−18.581	−0.682		<0.001
	Age	−0.361	−11.430	−0.497		<0.001
	BMI	0.106	3.230	0.160		0.001
	Total energy expenditure (kcal/day)	0.101	2.941	0.146		0.001
Model summary					0.615	<0.001
Knee endurance extension	Gender	−0.651	−24.225	−0.772		<0.001
	Age	−0.451	−17.658	−0.663		<0.001
	BMI	0.146	5.511	0.266		<0.001
	Total energy expenditure (kcal/day)	0.088	3.181	0.157		0.002
Model summary					0.748	<0.001

*BMI* body mass index

#### Upper body

Multivariate regression correlates of upper body muscle strength are displayed in Table 4. Factors accounting for 55.2 % of the variance in handgrip strength were gender (p < 0.001), age (p < 0.001), and time spent in light intensity physical activity (p < 0.001). Factors accounting for 77.2 % of the variance in isokinetic elbow flexion at 60°/s were gender (p < 0.001), BMI (p < 0.001), age (p < 0.001), and time spent in light intensity physical activity (p = 0.002). Season, test site, and VO_2max_ were not significant correlates of upper and lower body muscle strength. (p > 0.05).

**Table 4:** Multivariate models of correlates of upper body muscle strength among STOMP Participants (n = 412)

Muscle strength	Predictors	β	t	Partial r	r^2^	p
Handgrip strength	Gender	−0.715	−21.413	−0.731		<0.001
	Age	−0.187	−5.590	−0.269		<0.001
	Time in light intensity activity (min/day)	0.127	3.784	0.186		<0.001
Model summary					0.552	<0.001
Isokinetic elbow flexion 60°/s	Gender	−0.824	−33.748	−0.861		<0.001
	BMI	0.155	6.307	0.301		<0.001
	Age	−0.145	−6.032	−0.289		<0.001
	Time in light intensity activity (min/day)	0.074	3.085	0.153		0.002
Model summary					0.772	<0.001

*BM* body mass index

### Paffenbarger physical activity questionnaire

#### Lower body

Multivariate regression correlates of lower body muscle strength and endurance are displayed in Table 5. Factors accounting for 53.0 % of the variance in isometric knee extension were gender (p < 0.001), age (p < 0.001), BMI (p < 0.001), and total self reported energy expenditure (p = 0.033). Factors accounting for 60.6 % of the variance in isometric knee flexion were gender (p < 0.001), age (p < 0.001), BMI (p < 0.001), and total self reported energy expenditure (p = 0.010). Season, test site, and VO_2max_ were not significant correlates of upper and lower body muscle strength. (p > 0.05).

**Table 5:** Multivariate models of correlates of lower body muscle strength among STOMP participants (n = 412)

Muscle strength	Predictors	β	t	Partial r	r^2^	p
Isometric knee extension	Gender	−0.589	−16.959	−0.645		<0.001
	Age	−0.354	−10.333	−0.457		<0.001
	BMI	0.178	5.073	0.245		<0.001
	Total self reported energy expenditure (MET*hr/wk)	0.074	2.145	0.106		0.033
Model summary					0.530	<0.001
Isometric knee flexion	Gender			−0.704		<0.001
	Age			−0.547		<0.001
	BMI			0.171		0.001
	Total self reported energy expenditure (MET*hr/wk)			0.127		0.010
Model summary					0.606	<0.001

*BMI* body mass index

#### Upper body

Multivariate regression correlates of upper body muscle strength are displayed in Table 6. Factors accounting for 55.4 % of the variance in handgrip strength were gender (p < 0.001), age (p < 0.001), BMI (p < 0.01), and total self reported energy expenditure (p = 0.062). Factors accounting for 72.0 % of the variance in isometric elbow extension were gender (p < 0.001), age (p < 0.001), BMI (p < 0.001), and total self reported energy expenditure (p = 0.051). Factors accounting for 72.0 % of the variance in isokinetic elbow flexion at 60°/s were gender (p < 0.001), BMI (p < 0.001), age (p < 0.001), and total self reported energy expenditure (p = 0.019). Factors accounting for 68.0 % of the variance in isokinetic elbow extension at 60˚/s were gender (p < 0.001), age (p < 0.001), BMI (p < 0.001), and total self reported energy expenditure (p = 0.045). Factors accounting for 67.9 % of the variance in isokinetic elbow flexion at 180˚/s were gender (p < 0.001), BMI (p < 0.001), age (p < 0.001), and total self reported energy expenditure (p = 0.062).

**Table 6:** Multivariate models of correlates of upper body muscle strength among STOMP participants (n = 412)

Muscle strength	Predictors	β	t	Partial r	r^2^	p
Handgrip strength	Gender	−0.697	−20.613	−0.716		<0.001
	Age	−0.201	−6.023	−0.287		<0.001
	BMI	0.093	2.720	0.134		<0.01
	Total self reported energy expenditure (MET*hr/wk)	0.062	1.868	0.093		0.062
Model summary					0.554	<0.001
Isometric elbow extension	Gender	−0.679	−23.089	−0.754		<0.001
	Age	−0.290	−9.979	−0.445		<0.001
	BMI	0.257	8.685	0.397		<0.001
	Total self reported energy expenditure (MET*h/wk)	0.57	1.954	0.097		0.051
Model summary					0.720	<0.001
Isokinetic elbow flexion 60°/s	Gender	−0.827	−34.042	−0.861		<0.001
	BMI	0.153	6.245	0.297		<0.001
	Age	−0.147	−6.124	−0.291		<0.001
	Total self reported energy expenditure (MET*hr/wk)	0.056	2.345	0.116		0.019
Model summary					0.720	<0.001
Isokinetic elbow extension 60°/s	Gender	−0.726	−25.353	−0.784		<0.001
	Age	−0.281	−9.938	−0.443		<0.001
	BMI	0.182	6.315	0.300		<0.001
	Total self reported energy expenditure (MET*hr/wk)	0.057	2.012	0.100		0.045
Model summary					0.680	<0.001
Isokinetic elbow flexion 180°/s	Gender	−0.774	−26.978	−0.802		<0.001
	BMI	0.152	5.274	0.254		<0.001
	Age	−0.141	−4.998	−0.241		<0.001
	Total self reported energy expenditure (MET*hr/wk)	0.053	1.875	0.093		0.062
Model summary					0.679	<0.001

*BMI*body mass index

In every model, gender was the strongest predictor of muscle strength (r = −0.861 to −0.645), followed by either age (r = −0.547 to −0.241) or BMI (r = 0.134–0.397), and last physical activity as measured by the Paffenbarger physical activity questionnaire (r = 0.093–0.127). Season, test site, and VO_2max_ were not significant correlates of upper and lower body muscle strength (p > 0.05).

## Discussion

The primary purpose of this STOMP sub-study was to provide insight into discrepant reports in the literature on the relationship between physical activity and muscle strength across the lifespan. Accordingly, we assessed the relationships among self-report and objective measures of habitual physical activity as they correlated with upper and lower body measures of muscle strength, as well as other factors that have been reported to influence these relationships, among a large cohort of approximately equal numbers of healthy, men and women from 20 to 91 years. Overall, the strongest correlates of upper and lower body muscle strength were gender accounting for 40–74 %, age 6–44 %, and BMI 2–16 % of the variance; whereas, physical activity correlations were much weaker explaining 1–3 % of the variance. Of note is that season, test site, and VO_2max_ did not emerge as significant covariates in these models (p > 0.05). Last, self-report measures of physical activity correlated more strongly with upper body strength measures, while objective measures of physical activity correlated more strongly with lower body strength measures. Our study demonstrated that the contributions of gender, age, and BMI in explaining the individual variability in muscle strength superseded those of physical activity. They also suggest that habitual physical activity may not be effective to reduce age and disease related declines in muscle strength (American College of Sports Medicine, 2013), p 210.

It has been well documented that men are stronger than women (Musselman and Brouwer 2005), muscle strength declines with age (Rogers and Evans 1993), and absolute muscle strength increases with body size (Hortobagyi et al. 1990). However, this is the first study to the best of our knowledge, to examine the contribution of each of these factors individually in conjunction with self- report and objective measures of habitual physical activity in explaining the variability in muscle strength and endurance among a large group of healthy men and women. Presently, the literature is mixed regarding the relationship between physical activity and muscle strength partially due to the methods used to assess physical activity and the population examined (Bryant et al. 2007; Daly et al. 2008; Forrest et al. 2007; Gerdhem et al. 2008; Jakobsen et al. 2010; Morie et al. 2010; Paalanne et al. 2009; Rantanen et al. 1997; Sandler et al. 1991). We found that the contributions of gender, age, and BMI were significantly greater than those of physical activity, regardless of the method of physical activity assessment; findings that further lend insight into reasons for mixed reports in this literature.

Consistent with our findings, a number of investigative teams have found self-reported physical activity to be associated with isometric or isokinetic muscle strength of the upper or lower body in adults across the lifespan (Forrest et al. 2007; Jakobsen et al. 2010; Paalanne et al. 2009; Rantanen et al. 1997; Sandler et al. 1991). Amongst these studies, only one (Sandler et al. 1991) examined additional factors that may have contributed to variations in muscle strength as measured isometrically via custom made devices for the extremities and isokinetically via the Cybex for trunk musculature, and found age was the strongest predictor of muscle strength, followed by either physical activity or body weight. The population studied by Sandler et al. (Sandler et al. 1991) included 620 healthy women from 25 to 55+ years, but their findings cannot be generalized to men. In contrast to these findings and ours, other studies have reported no correlation between self-reported physical activity and any measure of muscle strength among samples that included older adults age 50+ years (Bryant et al. 2007; Daly et al. 2008) suggesting that the older age of the populations in these studies may have obscured the influence of physical activity on muscle strength.

A unique aspect of our study is that we used both self-report and objective measures of physical activity to profile the multidimensional nature of physical activity (D. Thompson, Peacock, Western, and Batterham 2015). We found similar weak associations using both a self-report questionnaire and an accelerometer to measure physical activity and muscle strength (r = 0.139–0.186); findings which contrast the small body of literature utilizing an accelerometer to examine these relationships (Gerdhem et al. 2008; Morie et al. 2010). A possible explanation for the discrepancies between these findings and ours is that Gerdhem et al. (2008) and Morie et al. (2010) included only older adults of one gender (Gerdhem et al. 2008; Morie et al. 2010), whereas we examined both genders in healthy adults across the lifespan. In addition, the sample size of both of these other studies was small (n = 57–82) which may not have provided the power needed to capture the weak associations between physical activity and muscle strength that we found to be superseded by the stronger effects of gender, age, and BMI.

Another unanticipated finding of our study was that self-reported physical activity measured by the Paffenbarger Questionnaire correlated more strongly with measures of upper body strength, while physical activity measured with an accelerometer correlated more strongly with measures of lower body strength. A possible explanation for these differential associations of self-report versus objectively measured physical activity with muscle strength is an accelerometer worn on the hip, as opposed to self-reported physical activity, is most sensitive to recording movement in the vertical plane (Wolin, Heil, Askew, Matthews, and Bennett 2008). Movement in the vertical plane typically occurs more during lower body than upper body physical activities. Thus, our results support using multiple methods to assess the multidimensional aspects of physical activity (D. Thompson et al. 2015).

### Potential study limitations

There were limitations to this study. The study design of STOMP (P. D. Thompson et al. 2010) was not specifically designed to measure the relationship between physical activity and muscle strength among healthy adults across the lifespan. This STOMP sub-study was cross-sectional as opposed to a longitudinal intervention, and thus we can only comment on association rather than causation. The larger STOMP study was conducted among multiple sites that may have contributed to variations in procedures and equipment among the sites. Nevertheless, all sites followed a strict set of standard operating procedures, and monthly research meetings were held to monitor all progress to ensure consistency among the involved sites. Finally, the sample was not truly random; rather it was self-selected because only people who met the exclusion criteria and were willing to take a clinical drug for 6 months agreed to participate in the study.

### Study strengths

On the other hand, this study has several strengths. Perhaps one of the most important is that this study is distinguished from previous research by using multidimensional measurements of both physical activity and muscle strength. In addition, the sample size was large and encompassed all adult ages, as well as having equal numbers of men and women, allowing for sufficient statistical power to test for associations between physical activity and muscle strength, using gender, age, and BMI as covariates.

## Conclusions and practical applications

The major findings of this study were that physical activity explained only 1–3 % of the variance in muscle strength; whereas gender explained 40–74 %, age 6–44 %, and BMI 2–16 % of the variance. In total, these models explained 53–77 % of the variance in muscle strength. Second, self-reported physical activity correlated with more strongly with measures of upper body strength, while objective measures of physical activity correlated with more strongly with measures of lower body strength.

In addition to providing clinicians with an overall picture of the important contributors to muscle strength across the lifespan, our findings provide insight into the discrepancies in this literature. Of import is that health care and exercise professionals should consider using multiple measures of physical activity and muscle strength and carefully consider the age and gender of the populations they employ to assess these relationships in their studies. Future studies may consider investigating these relationships in disease populations, as the contribution of physical activity to muscle strength may differ significantly from the healthy adult population.

In summary, although physical activity correlations with muscle strength appear weak indicating that habitual physical activity may not be effective at reducing age and disease related declines in muscle strength, maintaining a physical active lifestyle is crucial to healthy aging due to its many health benefits (US Department of Health and Human Services 2008).

## Abbreviations

BMI: body mass index
STOMP: the effect of statins on skeletal muscle function
MET: metabolic equivalent task
